# Cutaneous Rosai–Dorfman disease involving the left ear responsive to systemic glucocorticoid: a case report

**DOI:** 10.3389/fmed.2026.1797006

**Published:** 2026-04-21

**Authors:** Xiaoxue Zhuo, Zhuoma Dangzeng, Peiyu Zhou, Tingting Wang, Lin Wang

**Affiliations:** Department of Dermatology, West China Hospital, Sichuan University, Chengdu, Sichuan, China

**Keywords:** case report, external auditory canal, Rosai-Dorfman disease, sinus histiocytosis with massive lymphadenopathy, systematic glucocorticoid

## Abstract

A 49-year-old man presented with a 1-year history of red facial nodules and a 3-month history of left-sided hearing decline. A biopsy revealed mixed inflammation and emperipolesis of histiocyte-like cells. An immunohistochemical analysis demonstrated that the histiocyte-like cells were positive for S100 and CD68_PGM1_ and negative for CD1a and CD207, confirming the diagnosis of cutaneous Rosai–Dorfman disease (CRDD). The initial treatment consisted of oral prednisone acetate combined with methotrexate. Due to limited efficacy and adverse effects, methotrexate was discontinued, and prednisone acetate monotherapy was used. After 2 months, the patient’s skin lesions gradually resolved, and hearing improved. This case demonstrates that CRDD involving the ear can lead to hearing recovery following systemic corticosteroid therapy.

## Introduction

Rosai–Dorfman disease (RDD) is a rare, benign, systemic histiocytic proliferative disorder primarily affecting lymph nodes. It is defined by the abnormal accumulation of S100-positive histiocytes that exhibit non-destructive emperipolesis in both nodal and/or extranodal sites (1, 2). Although RDD with skin involvement is common, pure cutaneous RDD (CRDD) is uncommon, and CRDD with auricular-canal involvement is exceptionally rare (3–6). In this study, we describe an unusual case of CRDD involving the external auditory canal that caused temporary conductive hearing loss, with good hearing recovery achieved after systemic glucocorticoid therapy.

## Case report

**Figure 1:**
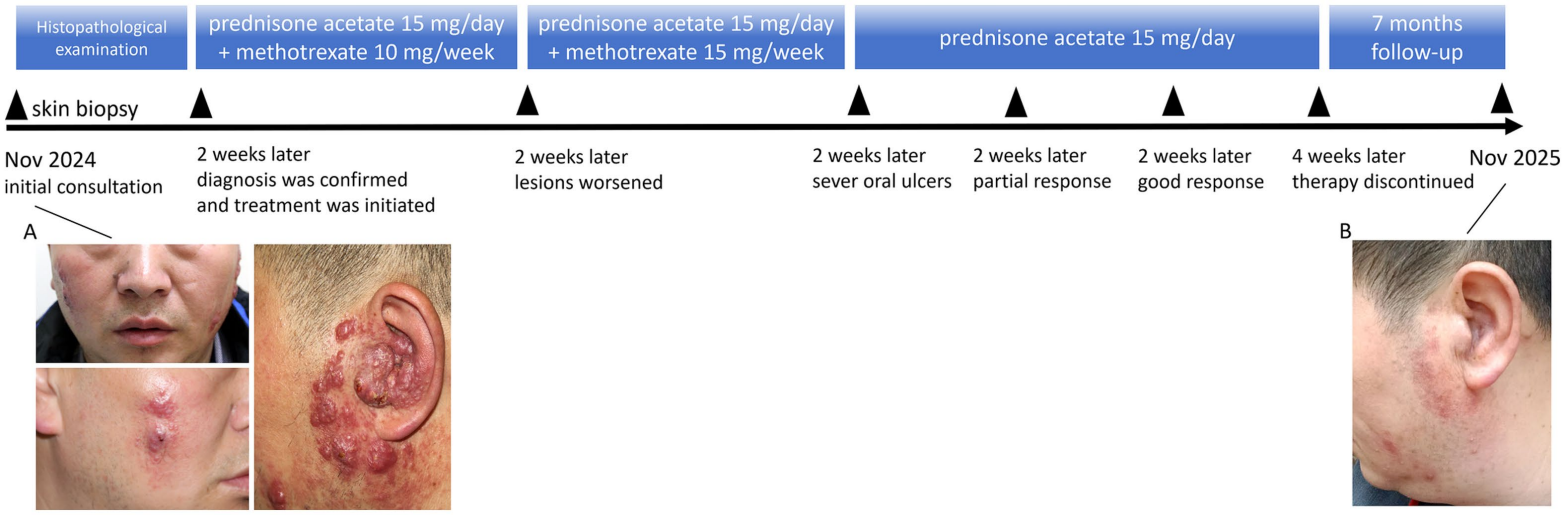
Timeline of disease, intervention, and outcome. **(A)** The morphological presentation of lesions at initial consultation (November 2024). **(B)** Resolution of ear plaque with systemic glucocorticoid (November 2025).

We present the case of a 49-year-old man with a 1-year history of red facial nodules and a 3-month history of left-sided hearing decline. Physical examination revealed red patches and nodules on both sides of the face and the left external auditory canal. The lesion partially occluded the canal, with no evidence of otorrhea (Figure 1A). The patient experienced no fever, weight loss, lymphadenopathy, night sweats, or other systemic symptoms, and the patient denied preceding trauma or infection. Laboratory evaluation, including complete blood count and comprehensive metabolic panel, showed no significant abnormalities. Otoscopic endoscopy revealed swelling in the left external auditory canal, and the middle and deep parts of the canal were normal. Regional lymph node ultrasonography showed slightly enlarged lymph nodes in the left cervical region with mildly abnormal partial architecture and enlarged lymph nodes in the bilateral inguinal regions suggestive of reactive hyperplasia. Chest CT revealed partial inflammatory changes, and abdominal ultrasonography showed hepatic steatosis.

**Figure 2:**
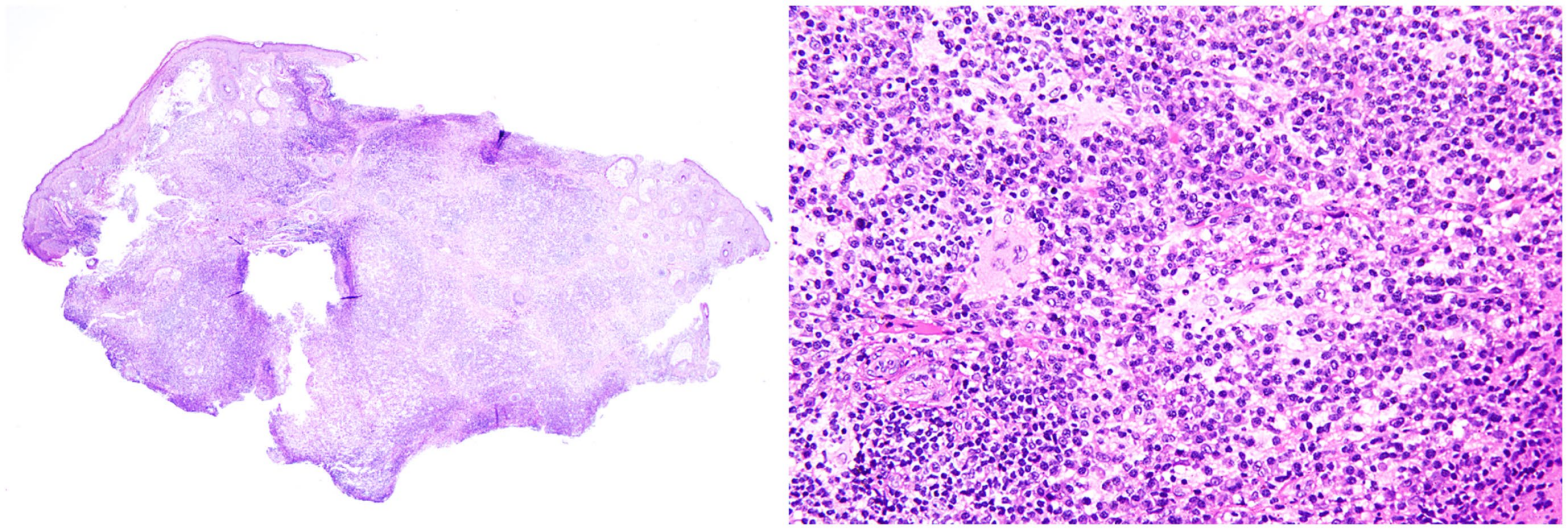
(Left) Low-power view of dermis showing sheet-like inflammatory infiltration with alternating dark and light zones (“starry-sky” pattern) (hematoxylin and eosin). (Right) High-power microscopic view (derived from the “starry-sky” infiltrate area in the dermis of the low-power view) of histiocyte-like cells with abundant and lightly stained cytoplasm and intracytoplasmic intact lymphocytes (emperipolesis) (hematoxylin and eosin).

**Figure 3:**
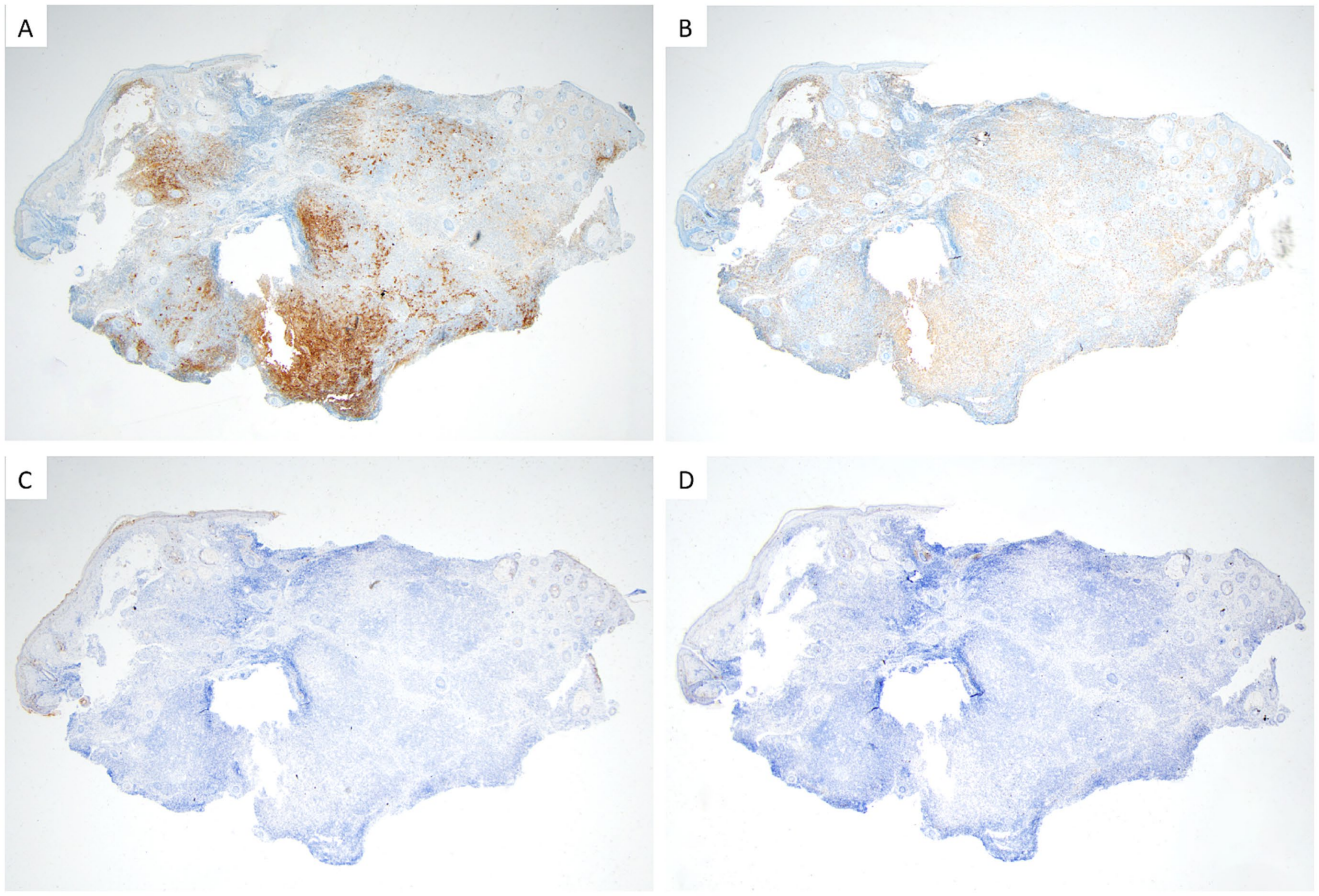
**(A)** Strong positive staining of S100. **(B)** Abundant distribution of CD68_PGM1_ positive staining. **(C)** Negative staining of CD1a. **(D)** Negative staining of CD207.

Histopathological examination showed epidermal atrophy and flattening, with mixed inflammatory cells infiltrating the dermis in sheets, including lymphocytes, epithelioid histiocyte-like cells, and some plasma cell. The cytoplasm of histiocyte-like cells was pale and abundant, and emperipolesis was observed (Figure 2). An immunohistochemical analysis demonstrated that the histiocyte-like cells were strongly positive for S100, CD68_PGM1_, CD163, Oct-2, and Bcl-2, partially positive for Cyclin D1, and negative for CD1a and CD207 (langerin) (Figure 3), confirming the diagnosis of CRDD.

The patient was initially treated with prednisone acetate 15 mg/day combined with methotrexate 10 mg/week. After 2 weeks of combination therapy, the lesions worsened, prompting an increase of methotrexate to 15 mg/week at week 3. Following 4 weeks of total treatment (2 weeks after methotrexate dose escalation), there was no significant improvement, and the patient developed severe oral ulcers, necessitating discontinuation of methotrexate. At this time point, no obvious response to therapy was observed. Prednisone acetate monotherapy (15 mg/day) was then maintained. After 2 weeks of monotherapy, the lesions gradually began to flatten. Hearing improvement was first documented 4 weeks after the initiation of prednisone monotherapy (8 weeks from treatment onset), concurrent with progressive flattening of the lesions and partial reopening of the left external auditory canal. Prednisone monotherapy was continued for an additional 4 weeks, during which the auricular and facial lesions flattened further and hearing returned to baseline. The patient remains asymptomatic, with stable hearing and no relapse during 7 months of follow-up (Figure 1B). The timeline is shown in Figure 1.

## Discussion

Rosai–Dorfman disease (RDD) was first described in lymph nodes in 1965 and was initially termed “sinus histiocytosis with massive lymphadenopathy (SHML)” by Rosai and Dorfman in 1969, emphasizing its characteristic clinical presentation (1). The typical clinical features of RDD include bilateral, painless cervical lymphadenopathy, with or without intermittent fever, night sweats, and weight loss. Histologically, it is characterized by the proliferation of S100-positive histiocytes and emperipolesis, defined as the active, non-destructive engulfment of lymphocytes, plasma cells, and erythrocytes by histiocytes (3, 7, 8). At the molecular level, approximately 30–50% of RDD cases harbor somatic mutations in the MAPK/ERK pathway, most commonly involving KRAS, NRAS, MAP2K1, and ARAF, with KRAS and MAP2K1 mutations typically being mutually exclusive (3, 9). In recent years, rare cases of RDD with BRAF-V600E mutations have also been reported (10, 11). Moreover, the prognosis for nodal cases is usually favorable. However, cases with systemic involvement, particularly those involving the kidneys, liver, or lower respiratory tract, tend to have a poorer prognosis (3). In 2016, the Histiocyte Society classified RDD within the “R group” of histiocytosis, while cutaneous RDD (CRDD) was recognized as a distinct disease entity belonging to class C histiocytosis (7). Clinically, CRDD typically presents as solitary or multiple asymptomatic yellow-to-reddish-brown papules, infiltrative plaques, or nodules, and may even manifest as tumor-like masses, with solitary lesions being more common (3, 12). CRDD occurs more frequently on the face and shows a female predominance (2, 13). The average age of CRDD onset is in the 40s (2, 8, 12). Patients with only CRDD have not been reported to progress to systemic disease and generally exhibit a self-limited course with a favorable prognosis (13).

CRDD involving the external auditory canal is exceptionally rare (5). The cases of RDD involving the ear are summarized in Table 1, based on a PubMed search. A total of 15 cases have been reported. The mean age was 32.6 ± 14.1, and the sex ratio (F/M) was 2.0. Nodular (26.7%) and swelling (26.7%) lesions were the most prevalent presentations. Hearing loss was noted in six (40.0%) cases. Combination therapy is mostly employed. Among reported cases, surgical intervention was the most common treatment method (used in seven patients), followed by systemic glucocorticoids (used in six patients). With the exception of 1 case that did not respond to antibiotics and another with an unknown outcome, the remaining 13 patients achieved either a good or partial response.

**Table 1:** Cases of Rosai–Dorfman disease involving the ear.

Patient age, y/sex	Presentations	Other symptoms	Treatment	Prognosis	References
39/F	Ulcerated firm mass in the external auditory canal	Chronic bloody otorrhea a, otalgia, disequilibrium	Aggressive surgery + topical antibiotics; Radiotherapy + cladribine	Initial treatment, no response; second treatment, good response; no relapse	Douleh et al. (29)
15/F	Erythematous firm nodules effacing the right external ear and canal	Tinnitus, hearing loss, and painless drainage	Systemic clofarabine + glucocorticoids	Good response	Kobayashi et al. (6)
48/F	Neoplasm at the external auditory meatus	Purulent discharge, conductive hearing loss, headache, low fever, weight loss	Surgery + systemic glucocorticoids	Good response, no relapse	Zhao et al. (30)
45/F	Ear redness/swelling in the cartilaginous portion and erythematous facial nodules	Scleritis, left inguinal and cervical lymphadenopathy	Systemic glucocorticoids + thalidomide	Good response	Ruan et al. (31)
37/F	Erythematous papules on the face and outer ears	Bilateral episcleritis, arthralgia	Topical tacrolimus, systemic glucocorticoids, dapsone, isotretinoin, rifampin, adalimumab, methylprednisolone, prednisone, cobimetinib	Good response after cobimetinib, no relapse after the cobimetinib withdrawal	López-Aldabe et al. (32)
22/M	Suppuration and swelling on the left ear	Low fever, hearing loss, hemorrhage, anemia; parotid and CNS masses; lymphadenopathy on MRI	Otologic surgery + Radiotherapy	Cured	Li et al. (23)
18/F	Suppuration and swelling on the right ear	Low fever, hearing loss, and hemorrhage; Otic, parotid, CNS masses; lymphadenopathy on MRI	Otologic surgery + Radiotherapy	Cured	Li et al. (23)
12/M	Tan/brown plaques of bilateral external auditory canals and middle ear with tracheobronchial tree involvement	Stridor, bilateral conductive hearing loss, and otitis media	Systemic corticosteroids + intermittent CO2/KTP laser excisions	Patial response	Ahsan et al. (24)
16/F	2 cm globular firm mass in the external auditory canal with nasopharyngeal involvement	Serosanguinous blood-stained discharge, snoring, and dyspnea	cladribine/cytarabine + local radiotherapy + steroids/6-mercaptopurine/methotrexate maintenance	Patial response	Arul et al. (33)
37/M	Red, tender, nodular, and indurated, non-scaly plaque covering the superior two-thirds of the pinna of the right ear	None reported	Topical/systemic corticosteroids + antibiotics; local intensity-modulated radiotherapy	Initial treatment, no response; second treatment, partial response	Bunick et al. (5)
20/M	Erythematous nodules effacing the left external ear and canal	Tinnitus, hearing loss, and headaches	Multiple antibiotics/antifungals + topical, intralesional, and systemic corticosteroids; complete auriculectomy with prosthetic ear placement	Initial treatments, no response; second treatment, good response	Lin et al. (4)
54/F	Symmetrical loss of normal ear contours, posterosuperior pinna thickening	No erythema or lymphadenopathy; mild tenderness	Corrective surgery	Good response, no relapse	Oo et al. (25)
37/M	Soft pink-violaceous plaque with a beaded border on the right pinna, with trunk/thigh involvement	Ear drainage	Refused radiologic investigations	Unknown	Hirt et al. (34)
49/F	Bilateral swelling of the ear helices with overlying red-brown nodules	Nasal bridge swelling, dyspnea	Subglottic nodule removal+ subcutaneous methotrexate	Partial response	Song et al. (35)
40/F	3 cm indurated nodule at the right tragal/preauricular region	None reported	Antibiotics	No response	Tsang et al. (36)

RDD has to be distinguished from other histiocytic disorders, especially Langerhans cell histiocytosis (LCH) and Erdheim–Chester disease (ECD), due to their clinical and molecular overlap. Mechanistically, all three are characterized by pathological ERK activation driven by somatic mutations in MAPK pathway genes (14). According to the 2016 Histiocyte Society classification, LCH and ECD belong to the “L group” (7). LCH has a broad spectrum of clinical manifestations, with characteristic presentations including lytic bone lesions, rash, and soft tissue swelling, and may also involve the ear (15). Histologically, LCH is characterized by histiocytes that are positive for S100, CD1a, and CD207, with coffee bean-shaped nuclei, and frequently harbors BRAF V600E mutations (7, 15). ECD typically presents with symmetric long bone involvement (sometimes showing “hairy kidney” sign on imaging), with skin involvement occurring in approximately 20% of cases, including the periorbital region, paranasal sinuses, and the ear (16, 17). Histologically, ECD is characterized by foamy histiocytes that are CD68- and CD163-positive but S100-, CD1a-, and CD207-negative, with BRAF mutations detected in approximately 57–70% of cases (18). In contrast, RDD is characterized by emperipolesis and shows positivity for S100 and CD68_PGM1_ and negativity for CD1a and CD207, with BRAF mutations being rare (3, 10, 11). Beyond these histiocytic disorders, clinical differential diagnosis should also include sarcoidosis, cutaneous lymphoma, and infectious granulomatous diseases, as they may present with morphologically similar skin lesions. Sarcoidosis is a diagnosis of exclusion, requiring histologic confirmation of non-caseating granulomas as well as polarization to exclude foreign bodies and tissue cultures to rule out infection (19). Cutaneous lymphoma demonstrates distinct clinicopathological features depending on the subtype. Tumor-stage mycosis fungoides (MF) presents with tumor nodules, whereas granulomatous MF may exhibit sarcoidal infiltrates or granuloma annulare-like features; both show epidermotropism of atypical T cells (20). Lymphomatoid papulosis manifests as recurrent erythematous papulonodular lesions with spontaneous regression, whereas primary cutaneous anaplastic large cell lymphoma presents as solitary or clustered brownish-violaceous nodules or tumors, with CD30 expression in more than 75% of neoplastic cells (21). B-cell lymphomas demonstrate dermal infiltrates of atypical B cells with clonal immunoglobulin gene rearrangements (20). Infectious granulomatous diseases show mixed inflammatory cell infiltrates pathologically and require the identification of causative organisms on special stains (22). These entities can be distinguished from RDD by the absence of emperipolesis and the lack of S100- and CD68-positive, CD1a-negative histiocytes on immunohistochemistry. Therefore, immunophenotypic and molecular differences are crucial for accurate diagnosis. When lesions involve the external auditory canal, the clinical differential diagnosis should also include granulomatous diseases, inflammatory pseudotumor, amyloidosis, relapsing polychondritis, and Hansen’s disease (23–25). Based on the clinical suspicion of multiple yellowish-red skin lesions and the typical pathological results, we have confirmed the diagnosis in our case. Familial RDD has been reported in association with patients with SLC29A3 gene mutations and may present with symptoms of hereditary sensorineural deafness (26). However, in our case, the patient’s hearing recovered as the external auditory canal lesions gradually resolved, indicating that the hearing impairment was unrelated to this diagnosis.

For patients with uncomplicated CRDD, observation is a reasonable approach, as 20–50% of patients experience spontaneous remission (27). In cases of unifocal CRDD, surgical excision is considered the most effective treatment (28). Other treatment modalities include corticosteroids (prednisone or dexamethasone), radiotherapy, chemotherapy, and various targeted therapies, with variable responses. A systematic review by Dhrif et al. reported that approximately 67% of patients with multifocal CRDD achieved complete remission following systemic corticosteroid therapy (2). In the present case, systemic corticosteroid therapy demonstrated remarkable efficacy. These findings indicate that, for patients with multifocal CRDD who fail to respond to conventional treatment, particularly those with lesions in anatomically sensitive sites, systemic corticosteroids may represent a viable treatment option.

Several limitations of this study should be acknowledged. Although ultrasound showed slightly enlarged left cervical lymph nodes, which differs from the massive bilateral cervical lymphadenopathy typical of systemic RDD, lymph node biopsy would be required to definitively exclude lymph node involvement. Second, prior methotrexate therapy may have had lasting effects, and its partial contribution to the observed outcomes cannot be completely excluded.

This case report emphasizes that CRDD involving the external auditory canal can cause temporary hearing loss, and excellent therapeutic outcomes, including full hearing recovery, may be achieved with systemic corticosteroid therapy. This observation provides valuable insights for early clinical diagnosis and treatment.

## Data Availability

The original contributions presented in the study are included in the article/supplementary material, further inquiries can be directed to the corresponding authors.

## Author’s note

Tingting Wang and Lin Wang jointly supervised this work.
